# Real-World Data on Bleeding Patterns of Hemophilia A Patients Treated with Emicizumab

**DOI:** 10.3390/jcm10194303

**Published:** 2021-09-22

**Authors:** Sarina Levy-Mendelovich, Tami Brutman-Barazani, Ivan Budnik, Einat Avishai, Assaf A. Barg, Tamara Levy, Mudi Misgav, Tami Livnat, Gili Kenet

**Affiliations:** 1 National Hemophilia Center, Sheba Medical Center, Tel Hashomer, Ramat Gan 52621, Israel; Sarina.Levy@sheba.health.gov.il (S.L.-M.); Tami.barazanibrutman@sheba.health.gov.il (T.B.-B.); einat.avishai@sheba.health.gov.il (E.A.); assaf.barg@sheba.health.gov.il (A.A.B.); mudi.misgav@sheba.health.gov.il (M.M.); tami.livnat@sheba.health.gov.il (T.L.); 2 Amalia Biron Research Institute of Thrombosis and Hemostasis, Sackler School of Medicine, Tel Aviv University, Tel Aviv 52621, Israel; tamara.levy@sheba.health.gov.il; 3 The Sheba Talpiot Medical Leadership Program, Sheba Medical Center, Tel Hashomer, Ramat Gan 52621, Israel; 4 Department of Pathophysiology, Sechenov First Moscow State Medical University (Sechenov University), 119991 Moscow, Russia; budnik_i_a@staff.sechenov.ru

**Keywords:** emicizumab, hemophilia, monitoring, hemarthrosis, bleeding

## Abstract

Emicizumab (Hemlibra™) is approved for prophylaxis of hemophilia A (HA) patients. The HAVEN studies addressed bleeding reduction in emicizumab-treated patients, but real-world data on bleeding patterns during emicizumab therapy are lacking. We aimed to compare the occurrence of breakthrough bleeding at different time points, starting from emicizumab initiation. This longitudinal prospective observational cohort study included HA patients (*n* = 70, aged 1 month to 74.9 years) that completed at least 18 months of follow-up in our center. We analyzed the number of spontaneous and traumatic bleeds during selected time points of the study (“bleeding periods”). The percentage of traumatic and spontaneous bleeding episodes was not significantly different among “bleeding periods” (*P =* 0.053 and *P* = 0.092, respectively). Most trauma-related treated bleeds resulted from either hemarthrosis (53%) or head trauma (33%). Spontaneous bleeding episodes were mostly hemarthroses (80%). Potential associations of the patients’ age, annualized bleeding rate before emicizumab treatment, and the presence of inhibitors with spontaneous bleed occurrence were analyzed with binomial logistic regression. The odds of bleeding while on emicizumab increased by a factor of 1.029 (*P* = 0.034) for every one year of age. Conclusions: Our real-world data revealed that the risk of bleeding persists, especially in older patients, despite therapy with emicizumab. These data may help clinicians in counselling their patients and in planning their management.

## 1. Introduction

Hemophilia A (HA) is a genetic X-linked severe bleeding disorder characterized by spontaneous or traumatic bleeding due to coagulation factor VIII (FVIII) deficiency. Patients may experience recurrent hemarthrosis, leading to severe joint damage at a young age [1]. Repeated intravenous (IV) replacement therapy infusions had been the backbone of prophylactic treatment for avoiding the vicious cycle of bleeding, inflammation, and arthropathy [2,3,4]. The concept of non-replacement therapy was recently introduced into hemophilia care, and several ongoing clinical trials are aimed at restoring hemostasis by rebalancing the coagulation factors and natural inhibitors [5].

Emicizumab (Hemlibra^®^, Roche) is a humanized IgG4 bi-specific antibody with affinity to factor IX/FIXa and factor X. It mimics the co-factor activity of FVIII by bridging the two factors [5,6,7,8]. The phase III clinical HAVEN studies, which were conducted in both adult and pediatric severe HA patients, have shown emicizumab to be safe and efficacious [9,10]. The drug is currently approved bythe US Food and Drug Administration and the European Medicines Agency for the treatment of HA patients with and withoutinhibitors [11]. In their pooled analyses of HAVEN 1–4 studies, Callaghan et al. [12] demonstrated an increase in the percentage of patients with zero treated bleeds throughout the study period (from 70.8% in weeks 1–24 to 83.7% in weeks 73–96). Real-world data that addressed the reduction in the annual bleeding rate (ABR) among emicizumab-treated patients are available [13,14,15], but comparable data on bleeding patterns during emicizumab therapy (specifically, spontaneous vs. traumatic, location of the bleed, and requirement of additional hemostatic therapy) are scarce. Moreover, there are none on the incidence of bleeding among HA patients during different time periods starting from the initiation of emicizumab therapy and continuing over time.

Our hemophilia treatment center longitudinally follows a large cohort of HA patients who are prophylactically treated by emicizumab. The aim of the current analysis was to describe breakthrough bleeding patterns (spontaneous as well as traumatic episodes) and analyze their occurrence at selected time points following emicizumab initiation.

## 2. Patients and Methods

The Israeli National Hemophilia Center treats ~700 patients, including 600 patients with HA. All severe HA (FVIII <1%) patients aged 1 month to 80 years treated with emicizumab were eligible for the current study. The center followed a cohort of 114 severe HA patients (age range 1 month to 74.9 years (median 14.6 years)) currently being treated with emicizumab. Only those who had been treated for at least 18 months were included, and our study group was composed of 70patients, including 28 patients (median age 5.6 years) with FVIII inhibitors (range, 0.5–900 BU at time of enrollment) and 42 patients (median age 17.2 years) without inhibitors. We divided the 18-month period of treatment into six shorter ones (“bleeding periods”) as follows: loading (weeks 1–4), and 1–3, 3–6, 6–9, 9–12, and 12–18 months. Bleeding episodes were documented separately for each “bleeding period” from the time of emicizumab prophylaxis initiation.

Emicizumab loading therapy of each study participant was initiated at our center, and the maintenance dose was either 1.5 mg/kg given once weekly or 3 mg/kg biweekly, according to protocol based upon HEAVEN studies [9,10]. The patients were instructed to contact and consult the center about any physical trauma, bleeding, or other adverse events. The study team made weekly telephone calls to each patient/family in order to follow therapy outcomes. After 1 year of follow-up, the telephone call frequency was reduced to once monthly. To compensate for variable times within the study follow-up, the ABR was calculated for each patient and the ABR for the year prior to initiation of emicizumab was compared to all traumatic and spontaneous bleeding episodes recorded during the study.

The study was approved by the Institutional Review Board (IRB) of Sheba Medical Center in accordance with the Declaration of Helsinki (protocol code 5858-19-SMC), and the patients or guardians of the participants provided informed consent.

### Statistical Analysis

Statistical analysis was performed with IBM SPSS Statistics (version 23.0; Armonk, NY, USA: IBM Corp.). Continuous variables were presented as median and interquartile range. Categorical variables were presented as counts and percentages. The Mann–Whitney *U* test was used to compare patient subgroups for continuous variables. The Chi-square test was used to compare patient subgroups for proportions. The Cochran’s *Q* test was used to compare the proportions of bleeding patients between different “bleeding periods” of the study (repeated measures proportions). Binomial logistic regression analysis was performed to investigate the relationship between the patients’ age, ABR, and the presence of inhibitors as predictor variables and the development of at least one episode of spontaneous bleeding during emicizumab treatment as a binary outcome variable. Two-tailed *P* values of less than 0.05 were considered statistically significant.

## 3. Results

**Figure 1:**
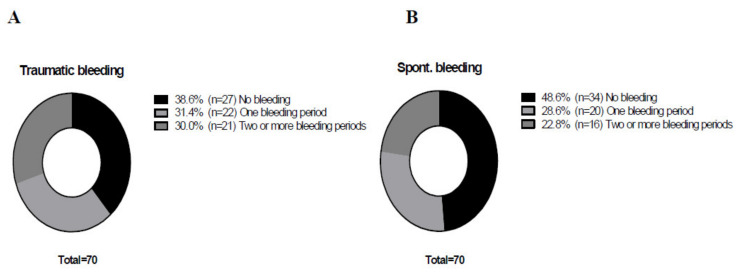
Proportion of patients experiencing either traumatic (**A**) or spontaneous (**B**) bleeding episodes throughout the study’s designated bleeding periods.

**Figure 2:**
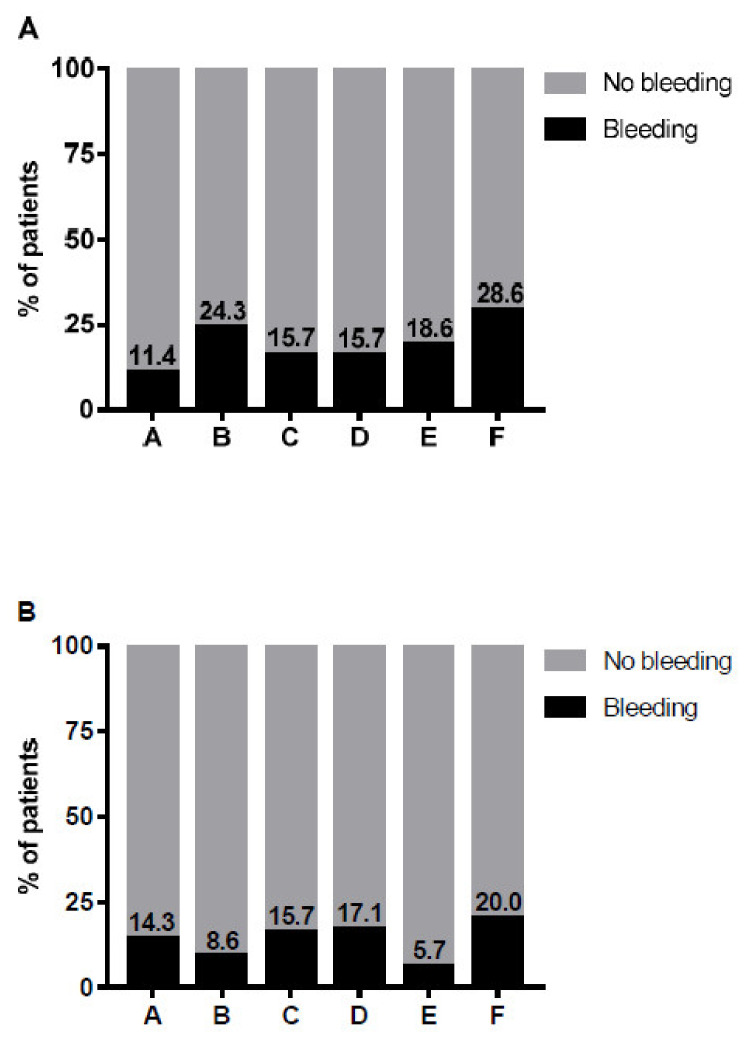
Proportion of patients with traumatic (**A**) and spontaneous (**B**) bleeding during emicizumab therapy. The proportions of bleeding patients at different “bleeding periods” of the study were compared using the Cochran’s *Q* test. No statistically significant difference in the proportions of patients with either traumatic or spontaneous bleeding episodes during emicizumab therapy was revealed (*P* = 0.053 and *P* = 0.092, respectively). “Bleeding periods”: A—loading, B—1–3 months, C—3–6 months, D–6–9 months, E—9–12 months, F—12–18 months. Black—% of patients with bleeding, gray bars—% of patients with no bleeds.

The patients’ demographic characteristics are presented in Table 1. Out of 70 HA patients, 36 (51%) had at least one episode of a spontaneous bleed and 43 (61%) had at least one traumatic bleed during the 18 months of follow-up. There was no significant difference between the proportions of patients that had either a traumatic or a spontaneous bleed during the various “bleeding periods” (*P* = 0.455) (Figure 1). There was no significant difference in the percentage of patients with traumatic and spontaneous bleeding episodes across six “bleeding periods” (*P* = 0.053 and *P* = 0.092, respectively) (Figure 2). Analysis of a subgroup consisting of 24 patients who had been treated for 24 months revealed that these patients experienced fewer spontaneous bleeds in the 18–24-month bleeding period, as compared to the 12–18-month bleeding period. Interestingly, the proportion of those patients who had experienced traumatic bleeds was not different compared to other treatment periods.

Among our 70 patients, a total of43 experienced trauma-related bleeding episodes, while 36 experienced spontaneous bleeds (seven patients had only trauma-related bleeding). The figure compares patients’ bleeding episodes during emicizumab prophylaxis. About a third of our patients experienced bleeding during one “bleeding period” only and the rest suffered bleeding during two or more bleeding periods. Proportions were compared using the Chi-square test, and no statistically significant difference between them was revealed (*P* = 0.455).

**Figure 3:**
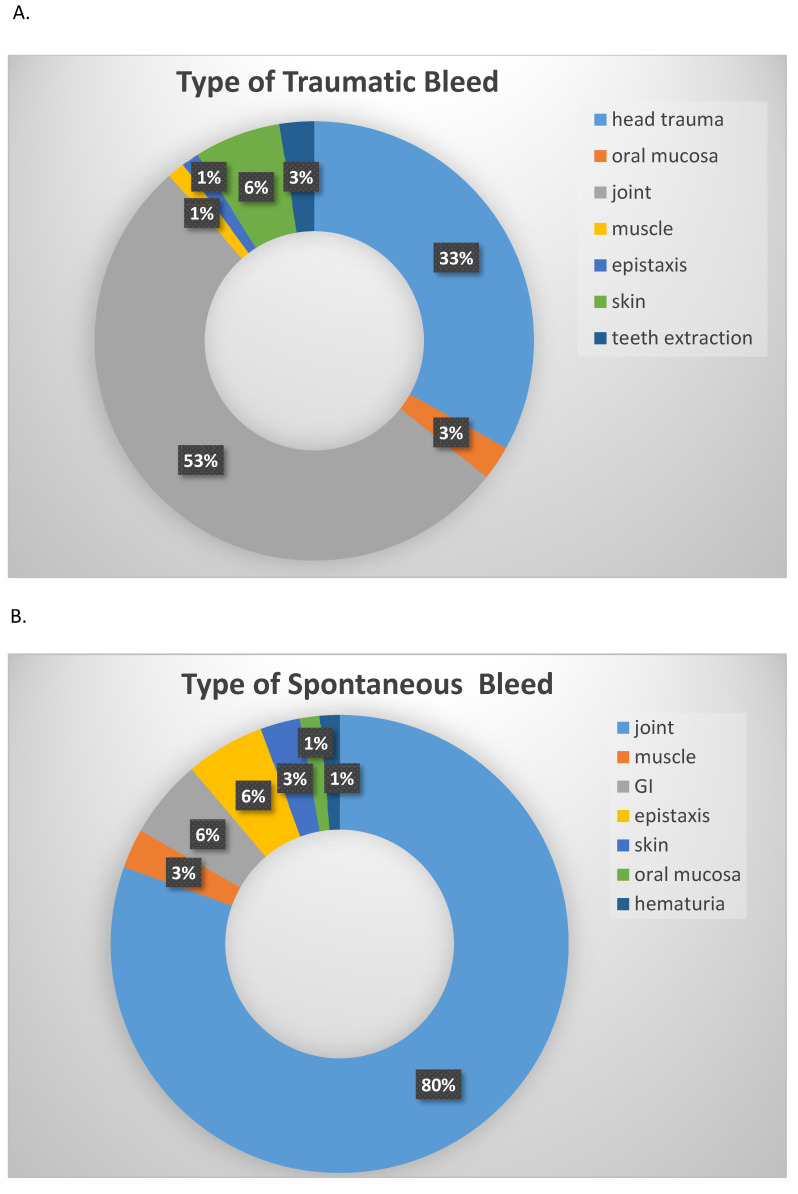
Location of traumatic and spontaneous bleeds. (**A**) Trauma-related bleeds. % of the different types of bleeds, each color is a different type of bleed. (**B**) Spontaneous bleeds. % of the different types of bleeds, each color is a different type of bleed.

**Figure 4:**
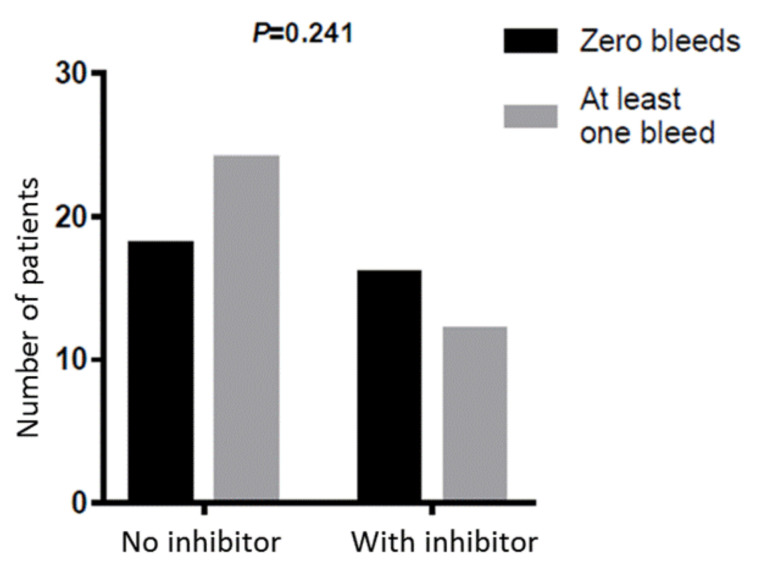
Comparison of spontaneous bleeding episodes in patients without vs. with FVIII inhibitors during 18-month emicizumab prophylaxis. The bleeding episodes were presented as counts. The proportions of bleeding episodes in either subgroup were compared using the Chi-square test. The resulting *P* value is shown in the graph.

**Figure 5:**
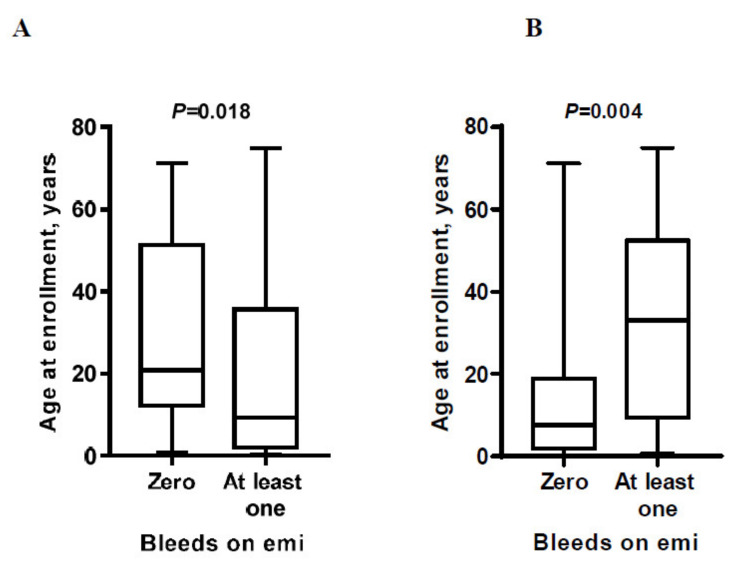
The association between age and the risk of traumatic (**A**) or spontaneous (**B**) bleeding episodes during 18 months of emicizumab prophylaxis. The data are presented as box-and-whisker plots. The boxes span the 25th to the 75th percentile, the line inside each box denotes the median, and the whiskers span the lowest to the highest observations. The Mann–Whitney *U* test was used to compare the patient subgroups. The resulting *P* values are shown in the graph. Patients with at least one traumatic bleed were statistically younger (**A**), whereas patients with spontaneous bleeds were statistically older (**B**).

Most of the trauma-related treated bleeds were either hemarthroses (53%) or head traumas (33%) (Figure 3A), as our cohort included a large group of pediatric patients (12 patients were younger than 2 years at study enrollment). Hemarthroses clearly prevailed among the spontaneous bleeding episodes (80%), and gastrointestinal bleeding and epistaxis were relatively common as well (6% each) (Figure 3B). The proportion of patients that had at least one traumatic bleeding episode was not significantly different between patients with vs. without FVIII inhibitors (*P* = 0.057), as was the proportion of patients that had at least one spontaneous bleeding episode (*P* = 0.241) (Figure 4). Interestingly, the patients with at least one traumatic bleed were younger than those without traumatic bleeds (*P* = 0.018), while the patients with at least one spontaneous bleed were older than those without spontaneous bleeds (*P* = 0.004) (Figure 5).

In order to investigate whether age, ABR before emicizumab treatment, and the presence of FVIII inhibitors are associated with the occurrence of spontaneousbleeding within 18-month follow-up of emicizumab prophylaxis, we performed a binomial logistic regression, and only age emerged as being statistically significantly associated with bleeding (Table 2). Specifically, the odds of bleeding increased by a factor of 1.029 (*P* = 0.034) for every one year of the patient’s age. In other words, being older was independently associated with an increased likelihood of at least one spontaneous bleeding episode within the 18-month period of emicizumab treatment.

## 4. Discussion

This is the first systematic real-world data cohort study to examine the incidence and pattern ofbreakthrough bleeding episodes and changes in breakthrough bleeding occurrence within different timeframes during emicizumab prophylaxis. Breakthrough spontaneous bleeding episodes were sustained in 51% of the treated patients, correlating with the findings of emicizumab studies on adults with HA with inhibitors [8,10,16]. As expected, we did not find any significant difference in the incidence of either traumatic or spontaneous bleeds during the selected time points during the first 18 months of follow-up given that their occurrence is not predictable. Unlike Callaghan et al. [12], we found no difference in the proportion of bleeding patients during the various “bleeding periods”. Such differences (e.g., bleeding reduction associated with target joint resolution) may have been perceived with a larger group of patients or with longer than 18 months’ follow-up. Notably, a reduction in spontaneous bleeds was apparent between 18 and 24 months of follow-up in a subgroup of our patients, in agreement with Callaghan et al. That finding may be explained by restrictions in mobility and fewer outdoor activities during the COVID-19 pandemic.

The presence of FVIII inhibitors did not correlate with a higher incidence of spontaneous bleeding events. This can be explained by the majority of patients with inhibitors in our cohort having been children, given that most bleeding episodes among children are not spontaneous but rather trauma-induced [13]. We performed a binomial logistic regression in order to investigate whether age, the number of bleeds before emicizumab treatment, and the presence of inhibitors are associated with traumatic bleeding within 18 months of follow-up and the results did not reach a level of significance since none of the independent variables included in the model could predict traumatic bleeding. The same analysis of those three parameters in association with spontaneous bleeding revealed only older age as being significantly and independently associated with bleeding during the 18-month follow-up period.

Our findings correlate with previous publications that demonstrated lower rates of breakthrough bleeding in children [9,17,18]. As observed by Hanley et al. [19], the majority of bleeds in HA patients are into joints. Our results further support theirs by demonstrating that 80% of spontaneous bleeds were joint bleeds. Interestingly, all of the spontaneous joint bleeds of our patients occurred into target joints, which are defined as joints experiencing hemarthroses threeor moretimes in a period of 6 months [18] due to the vicious cycle of hemorrhage–synovitis. That vicious cycle in adult HA patients may lead to arthropathy that persists despite adequate hemostasis, and it is especially disabling in the aging population [15,20]. The pooled analysis of the HAVEN studies showed that a large proportion of patients did not bleed into previously existing target joints, and that some “lost” their target joint definition due to reduced ABR. Our real-world data, however, are unable to confirm that finding. A possible explanation may stem from the fact that although target joints still tend to bleed after emicizumab prophylaxis has been initiated, treated patients gain confidence and become more physically active and engage in higher-risk sport activities [8,18,21]. Such increased activity may either induce pain of the “problem joints” [21] that leads to treatment by coagulation concentrates, mistakenly interpreted as a “breakthrough bleed”, or, alternatively, it may lead to actual hemarthrosis since those joints are more susceptible to further injury.

The strengths of our study include the longitudinal prospective follow-up of our single-center cohort and the availability of real-world data. The limitations include the lack of information about untreated bleeds, the lack of imaging studies for confirming the presence of bleeds versus synovitis or chronic joint pain, and the fact that patient-reported outcomes were not electronically collected on a daily basis. Our study was not empowered to compare various treatment strategies for breakthrough bleeding episodes occurring in HA patients during emicizumab prophylaxis, nor did it include subsequent joint score analyses.

## 5. Conclusions

Our findings indicate that the risk of bleeding persists despite therapy with emicizumab, especially in HA patients with problem and target joints, in children who sustain trauma-related bleeds, and in older patients. These data may help clinicians in advising their patients and in planning their management.

## Figures and Tables

**Table 1:** Demographic characteristics of patients at study entry.

	without FVIII Inhibitors (*n* = 42)	with FVIII Inhibitors (*n* = 28)	*P* Value
Age (years)	17.2 (9.2–45.9)	5.6 (1.0–34.5)	0.009 ^a^
Prior prophylaxis	34 (81%)	14 (50%)	0.006 ^b^
ITI	-	10 (36%)	NA
Inhibitor (BU)	-	12.0 (3.6–19.8)	NA
History of FVIII inhibitors	7 (17%)	-	NA
ABR	4 (1–12)	6 (3–10)	0.152 ^a^

Data are expressed as number (%) or median (interquartile range). Prior prophylaxis was defined as administration of either FVIII or bypassing agents at least twice weekly, before emicizumab prophylaxis period. BU, Bethesda units; ITI, immune tolerance induction; History ofFVIII inhibitors, 7/42 patients underwent successful ITI and were fully tolerized; ABR, annualized bleeding rate; NA, not applicable; ^a^ Mann–Whitney *U* test; ^b^ Chi-square test.

**Table 2:** Factors associated with spontaneous bleeding during emicizumab prophylaxis (as assessed by binomial logistic regression analysis).

Independent Variable	B	SE of B	*P* Value	Odds Ratio	95% CI for Odds Ratio
Age, years	0.028	0.013	0.034	1.029	1.002–1.056
ABR	0.021	0.029	0.462	1.021	0.966–1.080
Presence of FVIII inhibitors	−0.358	0.529	0.499	0.699	0.248–1.971
Constant	−0.613	0.457	0.179	0.542	NA

ABR = annualized bleeding rate during emicizumab prophylaxis; χ^2^(3) = 9.567, *P* = 0.023; Nagelkerke R^2^ = 0.170.; B, non-standardized coefficient; SE, standard error; CI, confidence interval; NA, not applicable.

## Data Availability

Data were collected from hospital databases as elaborated in the Section 2.
